# Supplementary figures and images for: Size-dependent steady state saturation limit in biomolecular transport through nuclear membranes
Source: PLoS One. 2024 Apr 16;19(4):e0297738. doi: 10.1371/journal.pone.0297738 (PMC11020410; doi:10.1371/journal.pone.0297738)

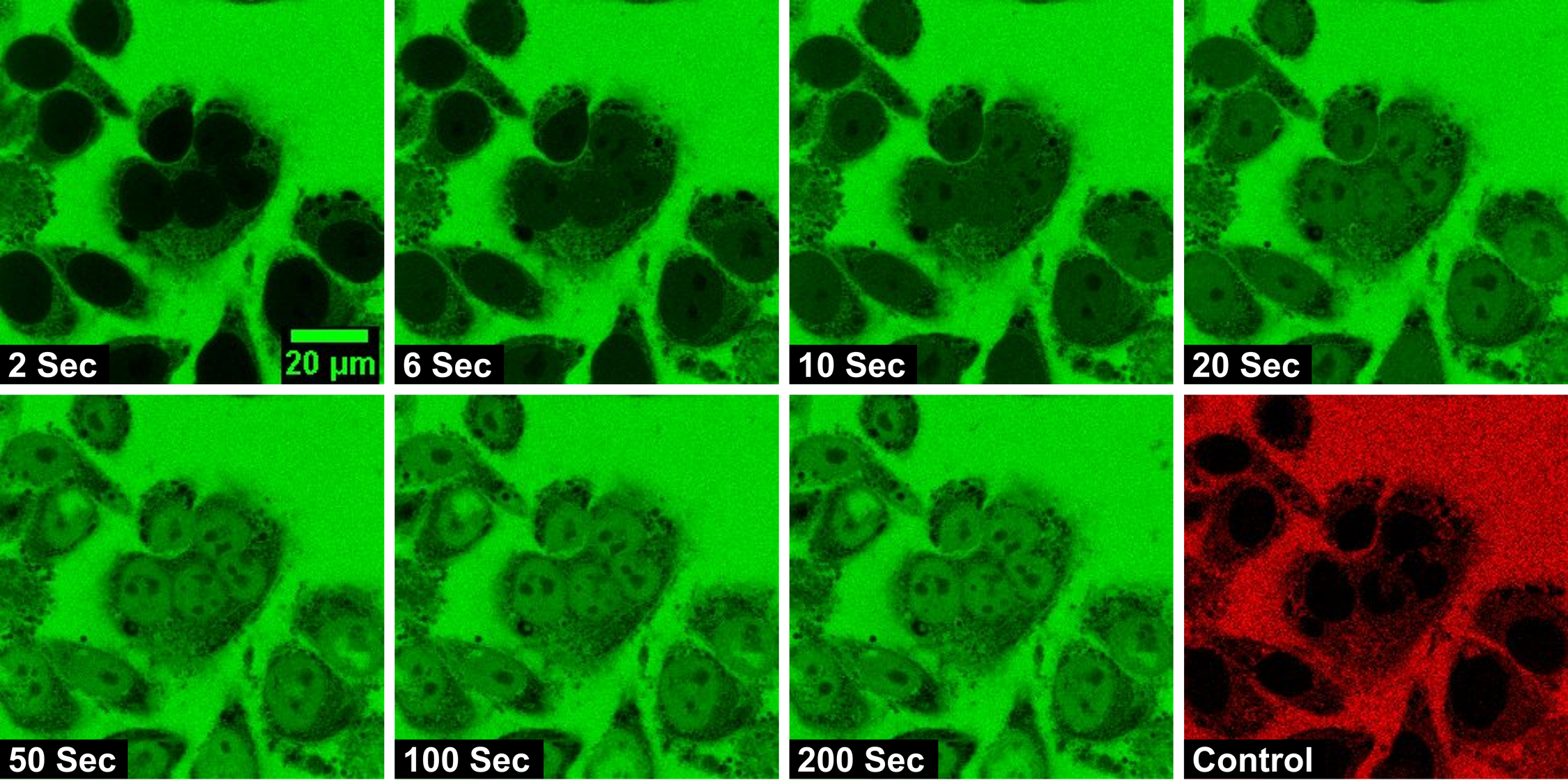

Supplement: S1 Fig — (TIF) [file pone.0297738.s001.tif]

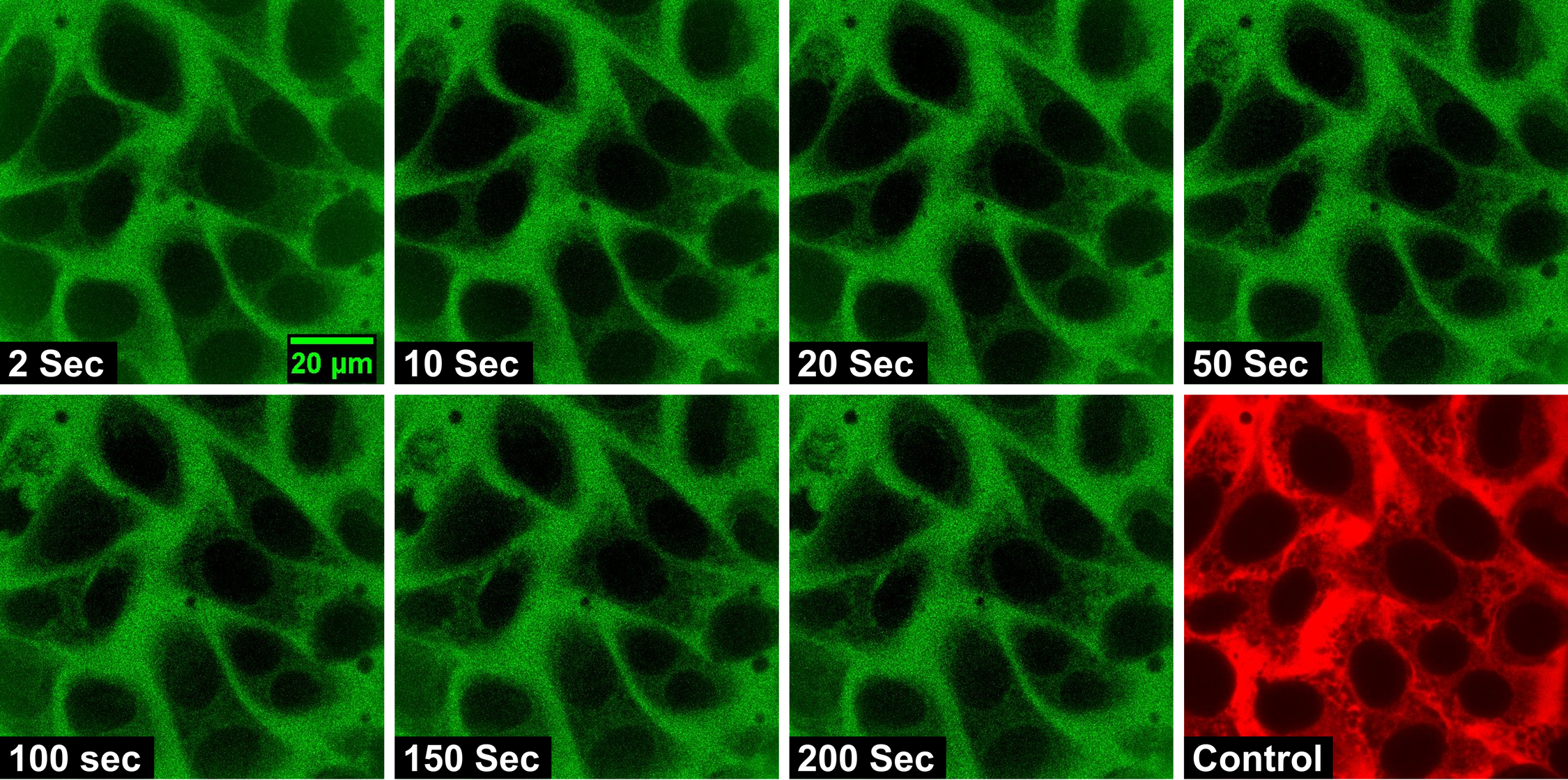

Supplement: S2 Fig — (TIF) [file pone.0297738.s002.tif]

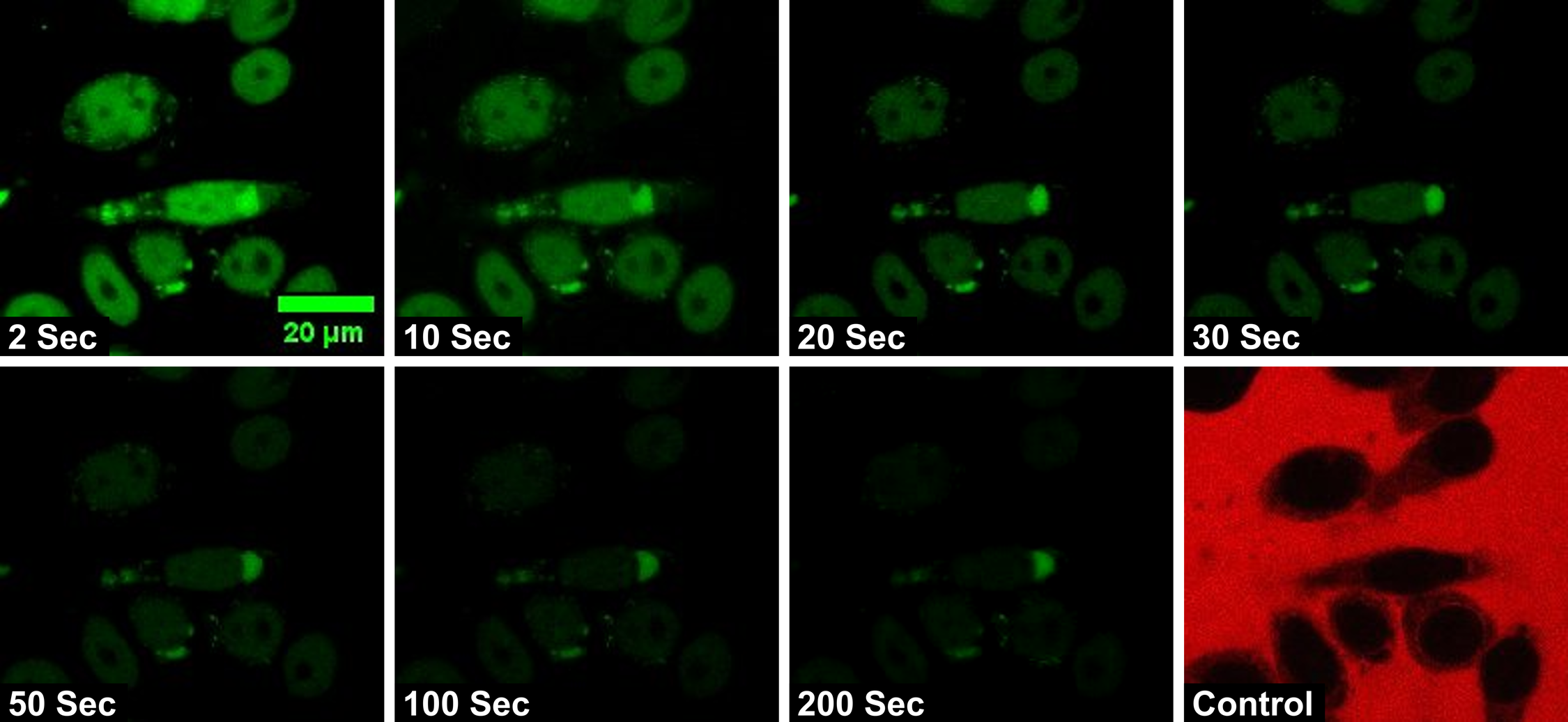

Supplement: S3 Fig — (TIF) [file pone.0297738.s003.tif]

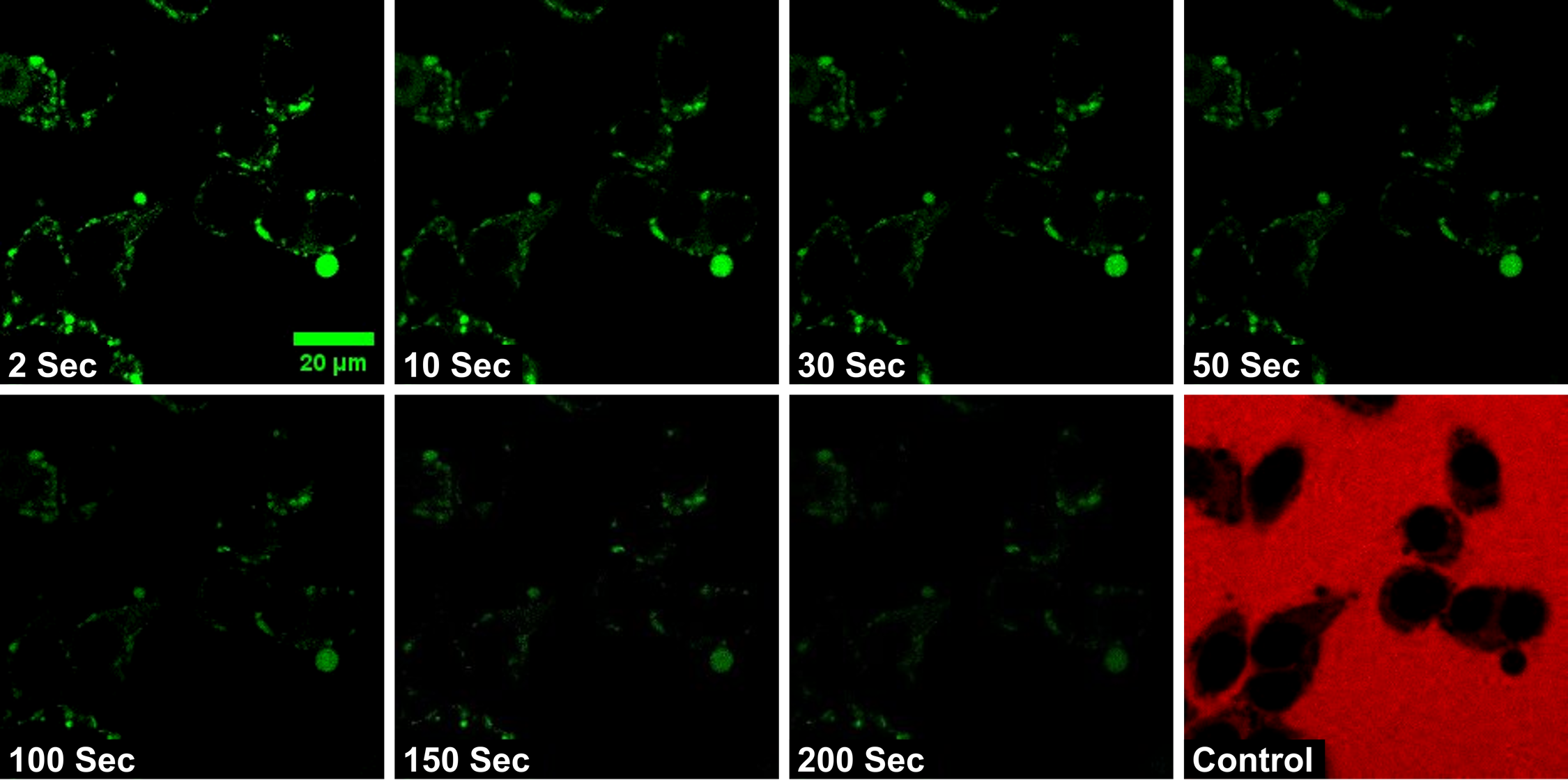

Supplement: S4 Fig — (TIF) [file pone.0297738.s004.tif]

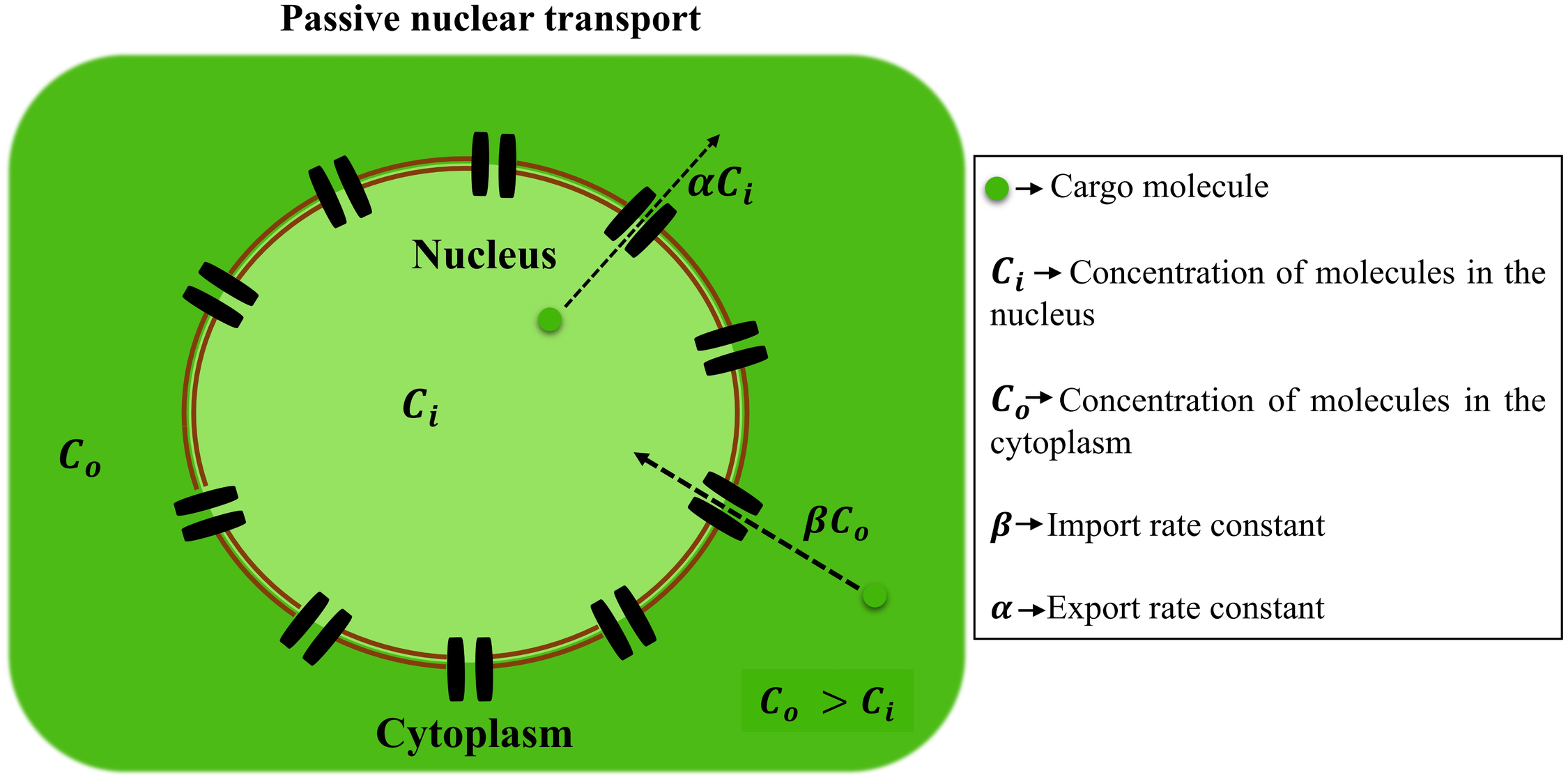

Supplement: S5 Fig — (TIF) [file pone.0297738.s005.tif]
